# Wild Bird Migration across the Qinghai-Tibetan Plateau: A Transmission Route for Highly Pathogenic H5N1

**DOI:** 10.1371/journal.pone.0017622

**Published:** 2011-03-09

**Authors:** Diann J. Prosser, Peng Cui, John Y. Takekawa, Mingjie Tang, Yuansheng Hou, Bridget M. Collins, Baoping Yan, Nichola J. Hill, Tianxian Li, Yongdong Li, Fumin Lei, Shan Guo, Zhi Xing, Yubang He, Yuanchun Zhou, David C. Douglas, William M. Perry, Scott H. Newman

**Affiliations:** 1 Patuxent Wildlife Research Center, United States Geological Survey, Beltsville, Maryland, United States of America; 2 Marine Estuarine Environmental Sciences, University of Maryland, College Park, Maryland, United States of America; 3 Institute of Zoology, Chinese Academy of Sciences, Beijing, China; 4 Graduate School, Chinese Academy of Sciences, Beijing, China; 5 Western Ecological Research Center, United States Geological Survey, Vallejo, California, United States of America; 6 Computer Network Information Center, Chinese Academy of Sciences, Beijing, China; 7 Qinghai Lake National Nature Reserve, State Forestry Administration, Xining, Qinghai, China; 8 Wuhan Institute of Virology, Chinese Academy of Sciences, Wuhan, Hubei, China; 9 Institute of Remote Sensing Applications, Chinese Academy of Sciences, Beijing, China; 10 Alaska Science Center, United States Geological Survey, Juneau, Alaska, United States of America; 11 EMPRES Wildlife Unit, Animal Production and Health Division, Food and Agriculture Organization of the United Nations, Viale delle Terme di Caracalla, Rome, Italy; Institut Pluridisciplinaire Hubert Curien, France

## Abstract

**Background:**

Qinghai Lake in central China has been at the center of debate on whether wild birds play a role in circulation of highly pathogenic avian influenza virus H5N1. In 2005, an unprecedented epizootic at Qinghai Lake killed more than 6000 migratory birds including over 3000 bar-headed geese *(Anser indicus)*. H5N1 subsequently spread to Europe and Africa, and in following years has re-emerged in wild birds along the Central Asia flyway several times.

**Methodology/Principal Findings:**

To better understand the potential involvement of wild birds in the spread of H5N1, we studied the movements of bar-headed geese marked with GPS satellite transmitters at Qinghai Lake in relation to virus outbreaks and disease risk factors. We discovered a previously undocumented migratory pathway between Qinghai Lake and the Lhasa Valley of Tibet where 93% of the 29 marked geese overwintered. From 2003–2009, sixteen outbreaks in poultry or wild birds were confirmed on the Qinghai-Tibet Plateau, and the majority were located within the migratory pathway of the geese. Spatial and temporal concordance between goose movements and three potential H5N1 virus sources (poultry farms, a captive bar-headed goose facility, and H5N1 outbreak locations) indicated ample opportunities existed for virus spillover and infection of migratory geese on the wintering grounds. Their potential as a vector of H5N1 was supported by rapid migration movements of some geese and genetic relatedness of H5N1 virus isolated from geese in Tibet and Qinghai Lake.

**Conclusions/Significance:**

This is the first study to compare phylogenetics of the virus with spatial ecology of its host, and the combined results suggest that wild birds play a role in the spread of H5N1 in this region. However, the strength of the evidence would be improved with additional sequences from both poultry and wild birds on the Qinghai-Tibet Plateau where H5N1 has a clear stronghold.

## Introduction

Highly pathogenic avian influenza (HPAI) subtype H5N1 (hereafter H5N1) first emerged in domestic geese of south-eastern China in 1996 [1], [2] and has since become endemic in poultry across much of Eurasia [3], [4]. Despite extensive eradication and vaccination campaigns, the virus continues to persist, re-emerging across much of its range. Waterfowl belonging to the family Anatidae (ducks, geese, and swans) are natural reservoirs for low pathogenic forms of avian influenza [5] and are rarely observed with HPAI infection [6]. H5N1 is unique, however, being the first HPAI virus to repeatedly cross the poultry species barrier back to wild birds. The first reports of spillover to wild birds occurred in low numbers of captive waterbirds at a Hong Kong waterfowl park in 2002 [7]. However, it was not until April 2005 that the first large epizootic (more than 6000 birds) occurred in wild species on the remote breeding grounds of Qinghai Lake (QHL), north-western China [8], [9]. Infections began in bar-headed geese *(Anser indicus)* soon after their migratory return to QHL, and were followed by infections in great black-headed gulls *(Larus ichthyaetus)*, brown-headed gulls *(Larus brunnicephalus)*, and great cormorants *(Phalacrocorax carbo)* 10 days later, and ruddy shelducks *(Tadorna ferruginea)* within 3 weeks [10]. Over half of the reported cases were in bar-headed geese. The QHL outbreak was significant not only because it was the first major H5N1 epizootic in wild populations, but also because it occurred in a region generally lacking poultry [11], which raised questions about the source of the virus and the ability of wild birds to transport virus over long distances. Following the QHL epizootic, H5N1 expanded beyond Asia, spreading northward and westward into Europe and Africa. All viruses isolated from these regions were subsequently traced back to QHL (clade 2.2) [4], [12] which fueled debate regarding the role wild birds play in H5N1 spread [13], [14], [15], [16].

QHL is a critical breeding ground and staging area for migratory waterbirds, supporting 150,000 migrants each year [17], including 15% of the global breeding population of bar-headed geese [18]. It is situated at the intersection of two major flyways: the Central Asian Flyway which extends from western Siberia through central Asia and south to India, and the East Asian Flyway which ranges from Russia through eastern China and south to Australia [19]. QHL holds multiple international designations of ecological importance: Important Bird Area [20], Key Staging Site for migrating Anatidae (waterfowl) [18], Wetland of International Importance [21], and national nature reserve of China. Despite its importance for migratory birds, little is known about wintering and breeding locations for species using the lake during different seasons [18].

If wild birds had played an integral role in transporting H5N1 to QHL, we hypothesized that the following conditions would be observed: (1) exposure to H5N1 virus on their wintering grounds or migratory stopovers during the northern hemispheric spring migration, (2) significant migration distances and viral shedding before becoming physiologically compromised, and (3) high sequence similarity between virus isolates from areas of exposure and those from QHL. To examine these questions, we sought to develop an improved understanding of the migratory movements, geographic range, habitat use, and overlap of bar-headed geese with poultry in zones of infection [18], [22], [23]. In 2007 and 2008, we tracked the migratory movements of 29 bar-headed geese from QHL using GPS satellite telemetry. We compared the spatial ecology of the host with the trajectory of H5N1 across the Qinghai-Tibet Plateau using phylogenetic analysis. This interdisciplinary approach used traditionally disparate tools – satellite telemetry and virological analysis - to assess the role of bar-headed geese in the transmission dynamics of H5N1.

## Materials and Methods

### Study Area

We sampled birds at the Qinghai Lake National Nature Reserve (QLNNR), Qinghai Province, in north-western China (36.82 N, 99.81 E). QHL is China's largest saltwater lake (4500 km^2^) and is located 3200 m above sea level on the eastern edge of the Qinghai-Tibet Plateau. The lake remains frozen November–March, and has a short rainy season from June–August (0.35 m annual average rainfall) [24]. The lake is a closed basin fed by 25 freshwater streams, the majority of which have intermittent flow [25].

### Marking and Virology Sampling

Bar-headed geese return to QHL in late March when temperatures are below freezing and the saline lake is still frozen. During this time, geese use freshwater springs and wetlands surrounding the lake before moving to one of three breeding colony sites in early April. We captured geese during the pre-breeding season at four non-colony sites to reduce disturbance to breeding colonies. Capture and marking occurred in late March 2007–2008 at QLNNR using monofilament noose sets and a net launcher (Coda Enterprises, Mesa, Arizona, USA). We recorded standard morphometrics (mass, flat wing chord, and diagonal tarsus [26]), age, and sex. Individuals were sampled for avian influenza (cloacal and tracheal swabs, blood serology) following standard procedures [27]. Virology samples were analyzed by the Chinese Academy of Sciences, Wuhan Institute of Virology with the following methods: (1) type A influenza with an ELISA test (Optical Density 630 above 0.23 as positive), (2) H5 subtype with RT-PCR [28], and (3) H5, H7, H9, and H10 antibodies with hemagglutinin inhibition [29].

Each bird was equipped with a 45-g, solar-powered GPS platform transmitter terminal (PTT; Microwave Telemetry, Columbia, Maryland, USA). PTTs measured 57×30×20 mm and were attached dorsally with a double-threaded backpack harness made of Teflon ribbon (Bally Ribbon Mills, Bally, Pennsylvania, USA). Transmitter packages weighed on average <2.1% of the bird's body mass. Capture, handling, and marking procedures were approved by the United States Geological Survey Patuxent Wildlife Research Center Animal Care and Use Committee and University of Maryland Baltimore County Institutional Animal Care and Use Committee (Protocol EE070200710).

The PTTs were programmed to take GPS locations every 2 hours, and data were uploaded to satellites every 2 days (CLS America Inc., Maryland, USA). We used ArcGIS 9.3 (Environmental Systems Research Institute, Inc., Redlands, California, USA) and Google Earth 5.0 (Google, Mountain View, California, USA) to plot and analyze telemetry locations.

### Telemetry, Movements, and H5N1 Risk Factors

We evaluated chronology, movement rates, seasonal habitat use, wintering locations, and migration stopover sites of the geese. Four seasons of the life cycle of the geese were defined: breeding (including post-breeding molt), fall migration, wintering, and spring migration. Migratory stopovers were defined as areas used during migration in which a goose moved no more than 20 km in a 24 h period. Stopover boundaries were drawn with minimum convex polygons (MCP) since we lacked enough locations to apply kernel home range methods. We calculated cumulative distance and time to complete each migration leg (the path between two consecutive stopover locations) for all geese that completed a minimum of one fall migration. The longest migration leg (km) for each goose was used to estimate the greatest distance travelled between stationary periods. Total migration distance for each individual was defined by the Euclidian distance between its northernmost and southernmost location. We used ArcGIS 9.3 and Hawth'sTools [30] to conduct these analyses.

Habitat use was evaluated by extracting land cover variables from telemetry locations according to season, pooled across individuals. Habitat use (expressed as a percentage) was based on the number of telemetry locations recorded at each land cover type divided by the total number of locations recorded in a season. Habitat features were derived from 1-km land-cover data produced by the Chinese Academy of Sciences [31], [32]. We reduced 25 land-cover classes into six for analysis: urban, cropland, grassland, wetland, woodland and other. Unlike conventional land cover data which represent the landscape as a single class per pixel, continuous fields in this dataset included the percent cover of all classes present within a pixel. Spatial overlap between goose movements and poultry farming was examined based on 1-km poultry densities from UNFAO Geonetwork [11].

#### Brownian bridge utilization distributions

To examine relationships between goose movements, landscape features, and potential risk factors associated with H5N1 transmission, we created Brownian Bridge utilization distributions (BBUD) [33] to describe goose migration patterns. Advantages of using the BBUD model over kernel density estimates for migration analyses is that the BBUD method avoids issues of serial correlation between points, assumes locations are temporally dependent, and explicitly includes length of time between locations in the model. This approach removes subjectivity in estimating temporal weights between intervals of unequal length and uses observed movements and measurable location error to model probability of occurrence. This effect is critical for migration when animals are moving long distances in short periods of time [33]. We created fall and spring migration BBUDs for each individual with Animal Space Use software [34]. An estimate of spring and fall migration routes were calculated as the mean probability of occurrence across individuals [33]. This required defining consistent seasonal dates across individuals from earliest departure and latest arrival dates of marked birds.

Fixed kernel home ranges were created for breeding and winter seasons. Least squares cross-validation was applied to obtain kernel smoothing parameters [35], [36] and utilization distribution models were developed for breeding and wintering with Animal Space Use [34]. Geese were weighted equally by including the same number of locations selected randomly for each individual. Probabilistic utilization distribution (UD) models for the group were created for all birds as a group with a Brownian bridge movement model for the spring and fall migration and fixed kernel home range models for breeding and non-breeding seasons.

#### HPAI H5N1 outbreaks

HPAI H5N1 outbreak data for 2003–2009 were obtained from two databases: the People's Republic of China Ministry of Agriculture Prevention and Control of Avian Influenza database [37] and the UNFAO's Emergency Prevention System for Transboundary Animal and Plant Pests and Diseases (EMPRES-i) database. Outbreak events were cross-checked between the two databases (Table S1) and imported into a GIS framework for analysis.

### Statistical Analyses

We conducted a geospatial analysis of bar-headed goose movements in relation to H5N1 risk factors and outbreak locations. This is the first effort we are aware of to compare separate analyses of risk factors for poultry and wild bird outbreaks. Following an information theoretic approach (AIC) to compare outbreak and non-outbreak (random) locations under two sets of *a priori* defined logistic regression models [38], we hypothesized that poultry outbreaks would be explained by anthropogenic factors (poultry density, etc.) and wild bird outbreaks by habitat and goose utilization distributions. Covariate predictors in the poultry models included latitude (Lat), bar-headed goose utilization distribution (BHGO UD), poultry density (PD), and cropland (Crop). Wild bird model covariates included Lat, BHGO UD, PD, grassland (Grass), and wetland (Wetl). Non-outbreak locations were represented by 10 random locations for each outbreak [39] and were drawn proportionally from the spatial extent of outbreaks in each season. A separate *a priori* univariate logistic regression (BHGO UD) was conducted to examine goose exposure to H5N1 outbreaks. Analyses were performed with the R statistical package [40] ‘glm’ function (family = binomial, link = −logit).

A classification and regression tree (CART) identified significant model covariates with the Rpart package in R [40], [41]. Binary trees were built by recursively partitioning explanatory variables into high and low categories that significantly contributed to the prediction of outbreak versus random locations [42].

Temporal aspects of outbreaks were examined for wild birds and domestic poultry by (a) comparing seasonal differences in numbers of outbreaks of each type (poultry or wild bird) and (b) comparing the observed and expected number of outbreaks based on the length of the season for each outbreak type using Fisher's Exact Tests [43], [44]. Expected numbers were calculated under the assumption that outbreaks were proportional to the number of days within a seasonal period.

### Phylogenetics

We conducted a phylogenetic analysis to determine genetic relatedness of H5N1 viruses isolated from wild birds and poultry with a focus on the Qinghai-Tibet Plateau. Sequences of HPAI H5N1 were obtained from the Genbank database hosted by the National Center for Biotechnology Information (NCBI) as of 30 September 2010. All phylogenetic analyses were performed with MEGA version 4.0 [45]. A total of 38 sequences of the HA (hemagglutinin) gene were trimmed to a length of 1550 bp and aligned with ClustalW following default settings. A phylogenetic tree was created by applying the neighbor-joining method [46] and evolutionary distances were computed with the Kimura 2-parameter method [47]. Bootstrapping (×1000) was used to assess the reliability of the tree topology. The tree was rooted to A/goose/Guangdong/1/96 and structured according to the World Health Organization system of avian influenza cladistics.

## Results

### Marking and Virology

We marked 29 bar-headed geese at QHL; 14 in 2007 and 15 in 2008 (Table S2) including 25 adults (14 male, 11 female) and 4 juveniles (2 male, 2 female). We obtained 20,150 Argos Doppler and 45,021 GPS locations (Table S2). Average transmitter lifespan was 10 months, but some PTTs performed for more than 2 years. GPS data was used for our analyses because they provided a large number of locations and lower spatial error compared with Argos data (15 m versus >100 m error). Twenty-two of the 29 marked geese were sampled for avian influenza virus (sampling materials were unavailable for the first seven birds marked). However, none of the sampled birds tested positive for avian influenza virus.

### Migration from Qinghai Lake

**Figure 1:**
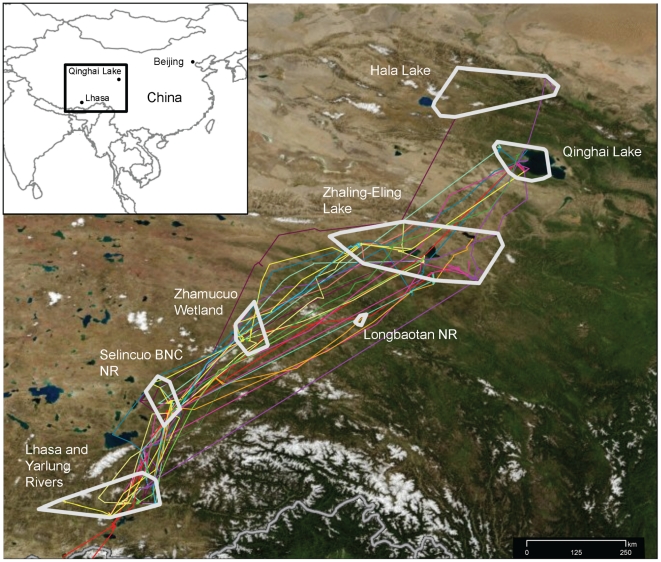
Migration routes, stopover locations, breeding, and wintering areas for bar-headed geese marked at Qinghai Lake. Migration routes are shown for the 15 individuals (each in unique color) that completed at least one fall migration. White polygons represent stopover areas. Individual 82081 (red path, full path in inset) wintered in India. Individuals 74898 (orange) and 82084 (yellow) are highlighted in Figure 3.

We defined the seasonal periods as: breeding (23 May–26 September; 126 days); fall migration (27 September–9 December; 74 days); wintering (10 December–5 March; 87 days); and spring migration (6 March–22 May; 78 days). We mapped migration routes, stopover sites, breeding locations, and wintering locations (Fig. 1).

#### Breeding

Twenty-seven geese remained at QHL through the breeding season while 2 moved farther north (Table 1): #82084 moved to Hala Lake and #67693 moved to Terkhiin Tsagaan Lake, Mongolia (within 200 km of H5N1 outbreaks in 2005, 2006, and 2009). Short movements (200–250 km) from the breeding grounds to molting locations occurred from 13 June to 19 September with most geese departing QHL in late June. The Zhaling-Eling Lake Region, a high-elevation (4290 m) wetland area 250 km southwest of Qinghai Lake was the most important post-breeding site (Table 1, Fig. 1).

**Table 1:** Breeding, post-breeding, spring and fall stopover, and wintering areas used by bar-headed geese captured at Qinghai Lake, 2007–2008.

Site Name	Distance (km) and Direction from Qinghai Lake	Latitude, Longitude (Degrees)	Time Period^a^	N^b^	Date Range^c^	Mean Length of Stay (range in days)	Number of Telemetry Fixes
Terkhiin Tsagaan Lake Region, Arkhangai Province, Mongolia	1240 N	48.09 N, 99.92 E	B07	1	5/7–6/5	29	49
Longbaotan Nature Reserve	570 SW	33.19 N, 96.47 E	B/PB 08	1^d^	5/22–10/23	154	222
Hala Lake Region	260 NW	38.13 N, 97.95 E	B08-09	2^d^	4/27–10/22	139 (103–178)	1,611
Qinghai Lake and Zhaling-Eling Lake Regions	0–350 SW	36.72 N, 99.02 E	B/PB 07–09	27^d^	3/25–11/4	154 (7–223)	19,338
Hala Lake Region	260 NW	38.13 N, 97.95 E	S08	1	5/13–5/13	1	3
Zhaling-Eling Lake Region	350 SW	34.93 N, 97.33 E	F07-08	7^d^	9/28–11/4	11 (2–23)	783
			S08-09	5	4/11–5/16	8 (2–29)	231
East of Wuli, Qumalai County	750 SW	34.63 N, 93.78 E	S09	1	4/8–5/8	31	131
Zhamucuo Wetland/Sanjiangyuan Nature Reserve	850 SW	33.09 N, 93.66 E	F07-08	10^d^	10/9–11/2	6 (1–11)	560
			S08	1	4/30–5/5	6	46
North of Xagquka, Biru County	970 SW	32.06 N, 92.86 E	S08	1	5/14–5/19	6	52
Selincuo Black-necked Crane Nature Reserve	1120 SW	31.65 N, 91.55 E	F07-08	9^d^	10/17–11/11	7 (5–14)	839
			S08-09	6	3/15–5/13	13 (3–38)	517
Namucuo Lake Region	1220 SW	30.50 N, 91.13 E	F07-08	7	10/22–11/17	13 (4–22)	808
			S09	2	3/14–4/18	9 (1–17)	178
Yamdrok Lake Region	1420 SW	28.84 N, 90.79 E	F08	2	11/2–12/7	18 (4–32)	309
			S09	1	3/11–4/7	28	229
Lhasa and Yarlung River Basins	1350 SW	29.40 N, 90.34 E	W07-09	15^d^	10/29–4/24	123 (17–175)	16,632
Bhitarkonika National Park, Orissa, India	2330 SW	20.65 N, 86.91 E	W08-09	1	12/9–3/6	87	1,001

a B = Breeding, PB = Post-breeding, S = Spring stopover, F = Fall stopover, W = Wintering area.

b N is the number of unique individuals that used a site in a given time period.

c Date ranges include arrival of first individual to departure of last individual.

d Includes individuals that returned during a second cycle, second visit included in tally.

#### Fall migration

Mean fall departure date was 13 October (range 27 September–30 October). Thirteen geese initiated their migration from Zhaling-Eling Lake region while 2 left from QHL. All geese followed similar routes: in general, they flew along a southwesterly path with stopover locations at Zhamucuo wetland, Selincuo Black-necked Crane Reserve, and Namucuo Lake (Table 1, Fig. 1).

#### Wintering

Geese arrived at wintering areas from 29 October–9 December (mean = 9 November). All birds wintered within 100 km of Lhasa except for #82081 which wintered south of the Himalaya at Bhitarkonika National Park in Orissa, eastern India. Lhasa wintering areas included the Pengbo River Basin (25 km northeast of Lhasa), the Lhasa River Basin (south of the cityflowing southwest for 50 km before meeting the Yarlung River), and the Yarlung River Basin (flowing eastward through Gongga then south becoming the Brahmaputra River) (Fig. 1).

#### Spring migration

The mean spring departure date for the geese was 1 April (range 6 March–April). Spring stopover locations included the Namucuo Lake region and Zhaling-Eling Lakes (Table 1, Fig. 1). The mean breeding arrival date was 29 April (range 28 March–22 May). Seven geese returned to breeding locations used the previous year (six to Qinghai Lake and one to Hala Lake). Two geese used different breeding locations in 2008: #74901 used Longbaotan wetland 570 km southwest of QHL; and #74902 used the Hala Lake region, 260 km northwest of QHL. The spring migration pathway (n = 9, orange line) was broader than the fall route (n = 15, yellow line) despite the fact that it was represented by fewer individuals.

#### Migration rates and stopover duration

Ninety-four migration legs were recorded (fall: 65 legs flown by 15 geese; spring: 29 legs flown by 9 geese). Migration was rapid – the longest leg (1158 km) was completed within 5.1 days (Table 2). Individual migrations included an average of two stopovers and were completed in <1 month (fall migration = 26 d; spring migration = 29 d). Migration rates (including time at stopovers) for the fall and the spring did not differ significantly (17 and 14 km/h, respectively; p = 0.23). Distance between QHL and the wintering grounds was 1300 km for 14 geese wintering near Lhasa and 2300 km for goose #82081 that wintered in India.

**Table 2:** Migration chronology and movement rates of bar-headed geese marked at Qinghai Lake during fall and spring migration, 2007–2008.

		Fall Migration				Spring Migration				Longest Leg^a^			
ID	Capture Date	Depart	Arrive	Length (d)	Stop-overs (N)	Depart	Arrive	Length (d)	Stop-overs (N)	Leg Distance (km)	Time (d)	Rate (km/d)	Total Displace-ment^b^ (km)
67582	3/25/07	10/10/07	11/13/07	35	3	4/7/08	4/22/08	16	2	534.1	1.2	457.8	1250
67690	3/25/07	10/10/07	11/3/07	25	2					293.6	1.3	234.9	1264
67695	3/25/07	10/12/07	11/1/07	21	2	4/24/08	5/10/08	17	2	815.5	5.8	141.8	1385
		10/9/08	10/29/08	21	2								
67698	3/31/07	10/24/07	10/31/07	8	1					716.7	2.6	277.4	1382
74898	3/30/07	10/28/07	11/3/07	7	1					500.2	1.1	461.7	1316
74900	3/31/07	10/30/07	11/13/07	15	1					571.5	0.8	685.8	1454
74901	3/31/07	10/1/07	11/11/07	42	2	4/6/08	5/22/08	47	2	584.9	0.7	877.3	1233
		10/23/08	10/29/08	7	1								
74902	3/30/07	10/24/07	11/17/07	25	2	4/8/08	5/13/08	36	2	981.6	1.6	620.0	1239
82079	4/2/08	10/20/08	11/12/08	24	1	3/25/09	3/28/09	4	0	1158.4	5.1	227.9	1235
82080	4/2/08	9/30/08	11/5/08	37	4	3/24/09			1	708.0	2.3	314.7	1329
82081	4/1/08	10/14/08	12/9/08	57	3	3/6/09	4/16/09	42	3	1036.0	4.3	239.1	2336
82082	3/30/08	9/30/08	11/15/08	47	4	4/1/09	4/27/09	27	2	780.1	2.3	346.7	1285
82084	3/30/08	10/22/08	11/9/08	19	2	4/5/09	5/21/09	47	2	642.8	2.1	308.5	1270
82085	3/30/08	9/27/08	11/6/08	41	4					342.6	1.1	316.2	1362
82086	3/31/08	10/15/08	10/29/08	15	1	3/14/09	4/9/09	27	2	950.7	4.9	193.4	1252

a Longest flight between two consecutive stationary areas.

b from northern-most to southern-most points.

### HPAI H5N1 outbreaks on the Qinghai-Tibet Plateau

**Figure 2:**
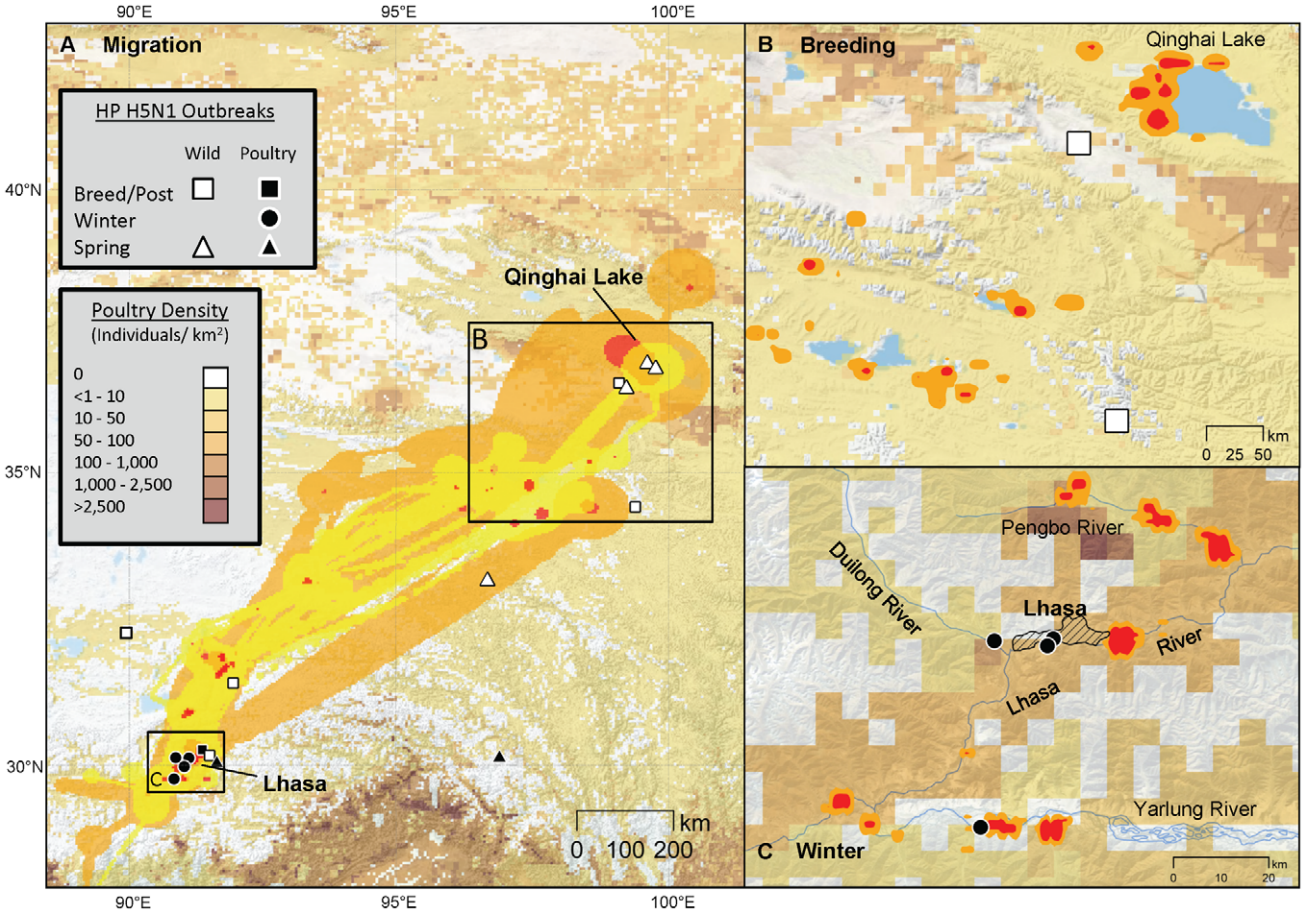
Brownian bridge utilization distributions in relation to poultry density and HPAI H5N1 outbreaks. Brownian bridge utilization distributions (A) describe fall (yellow; 27 Sep–9 Dec) and spring (orange; 6 Mar–22 May) goose migrations. Fixed kernel home ranges depict (B) population level breeding and post-breeding areas (C) and wintering areas, with only locations near outbreaks shown. Brown shading indicates poultry densities. H5N1 outbreak events in wild birds (white) and poultry (black) are indicated for 2003–2009. Two shading levels indicate isopleths containing 95% (red) and 99% (yellow-orange) of total locations.

Sixteen outbreaks were reported on the Qinghai-Tibet Plateau from 2003–2009; nine in wild birds and seven in poultry (Table S1). Fourteen outbreaks (87.5%) were located within the BHGO UD (Fig. 2a). All poultry cases occurred near Lhasa, whereas wild bird outbreaks were clustered around QHL and between Lhasa and QHL (Fig. 2a). Poultry outbreaks occurred in chickens and wild bird outbreaks occurred in several waterbird species including bar-headed geese, brown-headed gulls, great black-headed gulls, great cormorants, ruddy shelducks and great-crested grebes (*Podiceps cristatus*). However, the bar-headed goose was the primary species infected during the outbreaks, both in the total numbers killed and the frequency of outbreaks.

#### Habitat use and overlap with poultry and captive-reared geese

**Figure 3:**
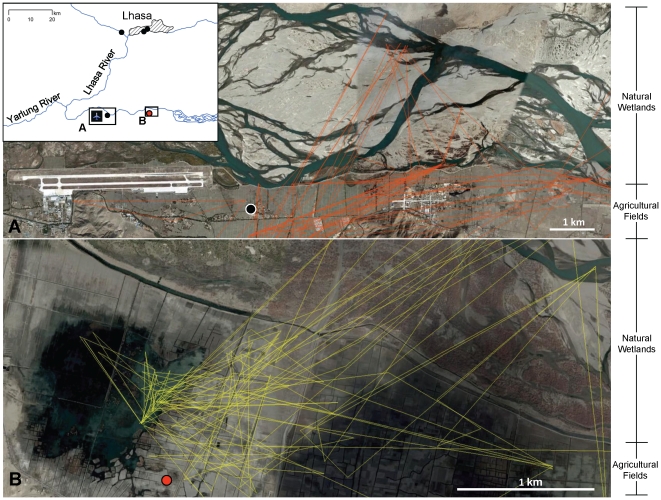
Concurrent use of natural wetlands and agricultural fields by wintering bar-headed geese near Lhasa. (A) Winter movements for goose 74898 (3 November 2007–2 April 2008; 1205 locations) in relation to a confirmed HPAI H5N1 outbreak in chickens on 21 January, 2008 (black circle). (B) Winter movements (9 November 2008–5 April 2009; 961 locations) for goose 82084 in relation to a captive bar-headed goose farm (red circle).

**Figure 4:**
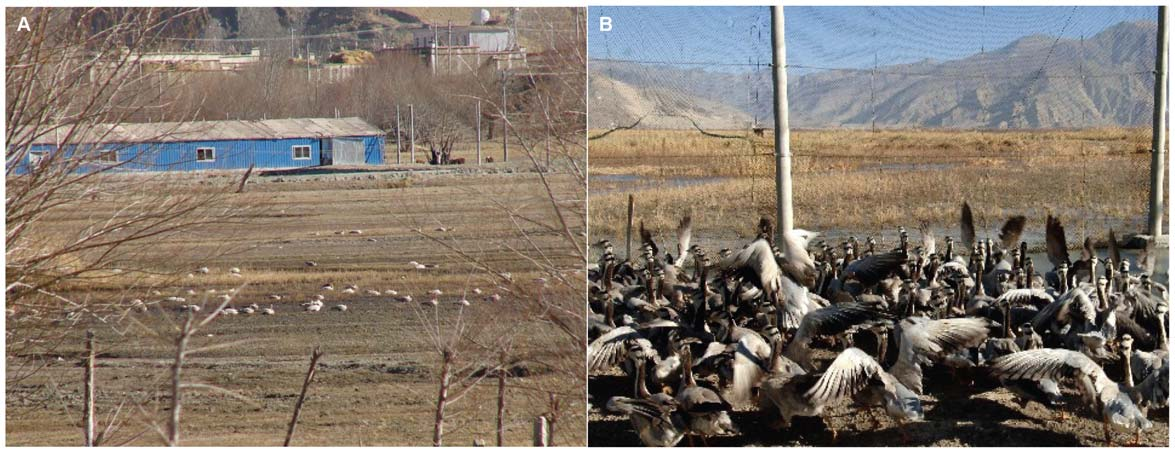
Bar-headed goose farming in Tibet. (A) Captive bar-headed geese in Gonggar County, Tibet as shown in a December 2007 China Tibet Information Center article (Wu 2007). (B) Wild bar-headed geese (foreground) shown in close proximity to a captive bar-headed goose farm (blue building in background) in a January 2007 photo from an anonymous source. Approximately 250 bar-headed geese were counted in outdoor net pens attached to the building (out of view in photo B). Approximate location of this farm is shown in a red circle (Fig. 3b).

Habitat use varied seasonally (Table 3). Breeding season habitats included natural wetlands (54% of locations) and grasslands (35%). Grasslands were primarily used during migration (78% fall, 77% spring). In the winter, a combination of natural wetlands (34%) and agricultural fields (39%) were used by the geese (Table 3, Fig. 3). Poultry densities at goose locations were near zero during all seasons except the winter which averaged 35.8 poultry head per km^2^ (Table 3). Potential exposure of geese to H5N1 occurred on the wintering grounds as was evidenced by their spatial and temporal overlap with a poultry outbreak in January 2008 (Fig. 3a) and with a bar-headed goose captive-rearing facility in 2009 (Fig. 3b and 4).

**Table 3:** Percent habitat type, poultry density, and human population densities at 43,841 satellite telemetry locations in China for 29 bar-headed geese marked at Qinghai Lake, 2007–2008.

	Percent Habitat Type							
Time Period	Crop	Grassland	Urban	Wetland	Woodland	Other Natural	Poultry Density (Indiv/km^2^)	Human Population (Indiv/km^2^)
Breeding/Post-breeding	0.2	34.9	0.0	53.6	0.1	11.1	0.6	0.7
Fall Migration	0.3	78.4	0.0	15.0	0.0	6.3	0.6	1.1
Spring Migration	0.0	76.6	0.0	12.3	0.0	11.1	0.5	1.5
Winter	38.5	25.0	0.2	33.6	0.8	1.9	35.8	134.9

#### Poultry outbreak risk factors

**Figure 5:**
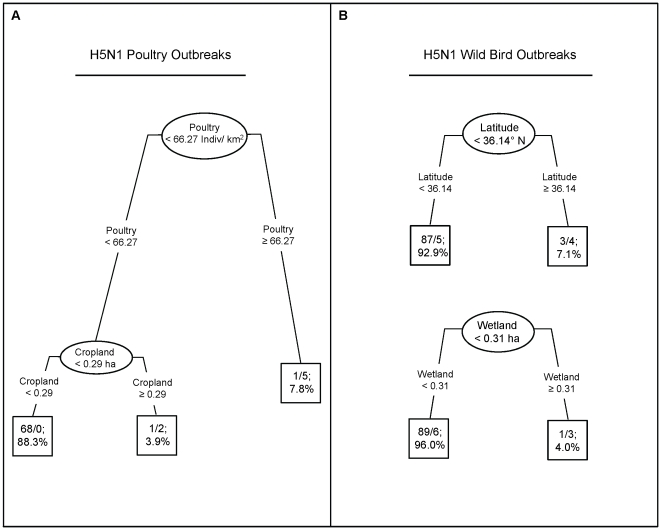
Classification and regression tree describing poultry (A) and wild bird (B) outbreaks on the Qinghai-Tibet Plateau. Numbers of random points (numerator), outbreak locations (denominator), and percentage of total sample are reported at each terminal node.

The AIC analysis for poultry outbreaks indicated two competing top models (Table 4): Model 1 included poultry density and cropland (AIC weight *w_i_* = 0.72) while Model 2 included poultry density, cropland, latitude, and BHGO UD (*w_i_* = 0.26). The top model indicated that domestic outbreak locations were explained by measures of poultry density and cropland area. The CART analysis separated outbreaks into those above 66 poultry per km^2^ and above 0.3 ha of cropland per km^2^ (Fig. 5a). Although the BHGO UD was not a strong indicator in predicting locations of poultry outbreaks, exposure of BHGO to H5N1 virus via outbreaks in poultry on the wintering grounds was confirmed (*a priori* univariate logistic regression, BHGO UD, p = 0.032).

**Table 4:** Akaike Information Criterion (AIC) best-fit model results for ten a priori models of domestic poultry and wild bird H5N1 outbreaks during 2003–2009 (n = 7 domestic, n = 9 wild) compared with random points drawn from minimum convex polygons per season (n = 70 domestic, n = 90 wild) on the Qinghai-Tibet Plateau.

Model	K	ΔAIC	Likelihood	*w_i_*
**Poultry Outbreaks** a				
PD + Crop	3	0.00	1.00	0.72
PD + Crop + Lat + BHGO UD	5	2.05	0.36	0.26
PD + Lat	3	9.34	0.01	0.01
PD + Lat + BHGO UD	4	9.80	0.01	0.01
PD	2	11.37	0.00	0.00
PD + BHGO UD	3	11.78	0.00	0.00
Crop	2	14.35	0.00	0.00
Lat	2	15.22	0.00	0.00
BHGO UD	2	21.92	0.00	0.00
null	0	25.07	0.00	0.00
**Wild Bird Outbreaks** b				
Wetl + Lat	3	0.00	1.00	0.70
Lat	2	2.29	0.32	0.22
Wetl	2	6.41	0.04	0.03
Wetl + PD	3	8.17	0.02	0.01
Wetl + Gras	3	8.31	0.02	0.01
Wetl + BHGO UD	3	8.36	0.02	0.01
null	0	9.47	0.01	0.01
Gras	2	10.83	0.00	0.00
PD	2	11.07	0.00	0.00
BHGO UD	2	11.44	0.00	0.00

a Covariates for poultry model were bar-headed goose utilization distributions (BHGO UD), poultry density (PD), latitude (Lat), and cropland (Crop).

b Covariates for wild bird models were BHGO UD, PD, Lat, grassland (Gras), and wetlands (Wetl).

K = number of model parameters, ΔAIC = AIC differences, *w_i_* = Akaike weights.

#### Wild bird outbreak risk factors

Two models best fit the data to explain locations of H5N1 outbreaks in wild birds on the Qinghai-Tibet Plateau. The top model (*w_i_* = 0.70) included latitude and wetlands while the second-ranked model (*w_i_* = 0.22) included latitude only (Table 4). CART results indicated differences at 36.14°N with wetland area larger than 0.3 ha (Fig. 5b). Contrary to our hypothesis, BHGO UD was not a significant predictor for wild bird outbreaks which tended to occur in remote regions. However, outbreaks were concentrated north of 36°N (near QHL), with fewer outbreaks located between QHL and Lhasa (Fig. 2a) where the BHGO UD is geographically broad and with lower use per pixel. In this region, the BHGO UD does not provide probabilities sensitive to predicting outbreak versus non-outbreak locations given the low number of total outbreaks. Thus, we conclude that while bar-headed geese are an important species in H5N1 events [8], [9], [10], [48], the BHGO UD is not a strong predictor across the Qinghai-Tibet Plateau, except near QHL where there are high concentrations of this species on the breeding grounds and greater number of outbreaks.

#### Timing of outbreaks

**Figure 6:**
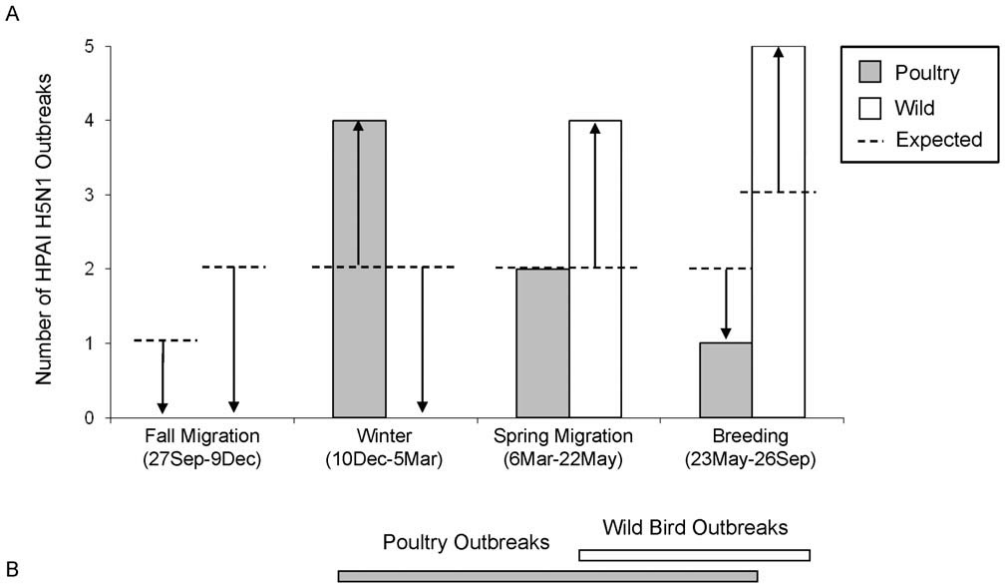
Comparison by season of wild and domestic outbreaks on the Qinghai-Tibet Plateau. (A) Observed versus expected values differed for both poultry and wild bird outbreaks when tested separately (Fisher Exact Test, poultry P = 0.079, wild P = 0.017). (B) Temporal distribution of wild bird outbreaks differed from domestic bird outbreaks (Fishers Exact Test, P = 0.008). Expected numbers were calculated under the assumption that outbreaks are proportional to the number of days within the seasonal period (Winter = 87 d, Spring Migration = 78 d, Breeding = 126 d, and Fall Migration = 74 d).

We found a temporal lag between peak seasons for poultry and wild bird outbreaks on the Qinghai-Tibet Plateau (Fisher's Exact Test, p = 0.008, Fig. 6a). Poultry outbreaks occurred during the winter, spring and breeding seasons, whereas wild bird outbreaks were not reported until the spring and breeding months (Fig. 6b, Table S1). More poultry outbreaks were found during the winter and the spring than expected (p = 0.079) and subsequently during the spring and the breeding seasons for wild birds (p = 0.017). In contrast, fewer outbreaks than expected occurred in the breeding and the fall for poultry, and the fall and the winter for wild birds (Fig. 6b). The first H5N1 outbreak reported on the Qinghai-Tibet Plateau occurred in poultry near Lhasa in February 2004, followed in the spring (Apr–Jun) of 2005 with the epizootic at QHL. Both results from Fig. 6 and timing of the first two outbreaks reported on the Qinghai-Tibet Plateau suggest initial outbreaks in poultry, followed by outbreaks in wild birds.

### Phylogenetics

**Figure 7:**
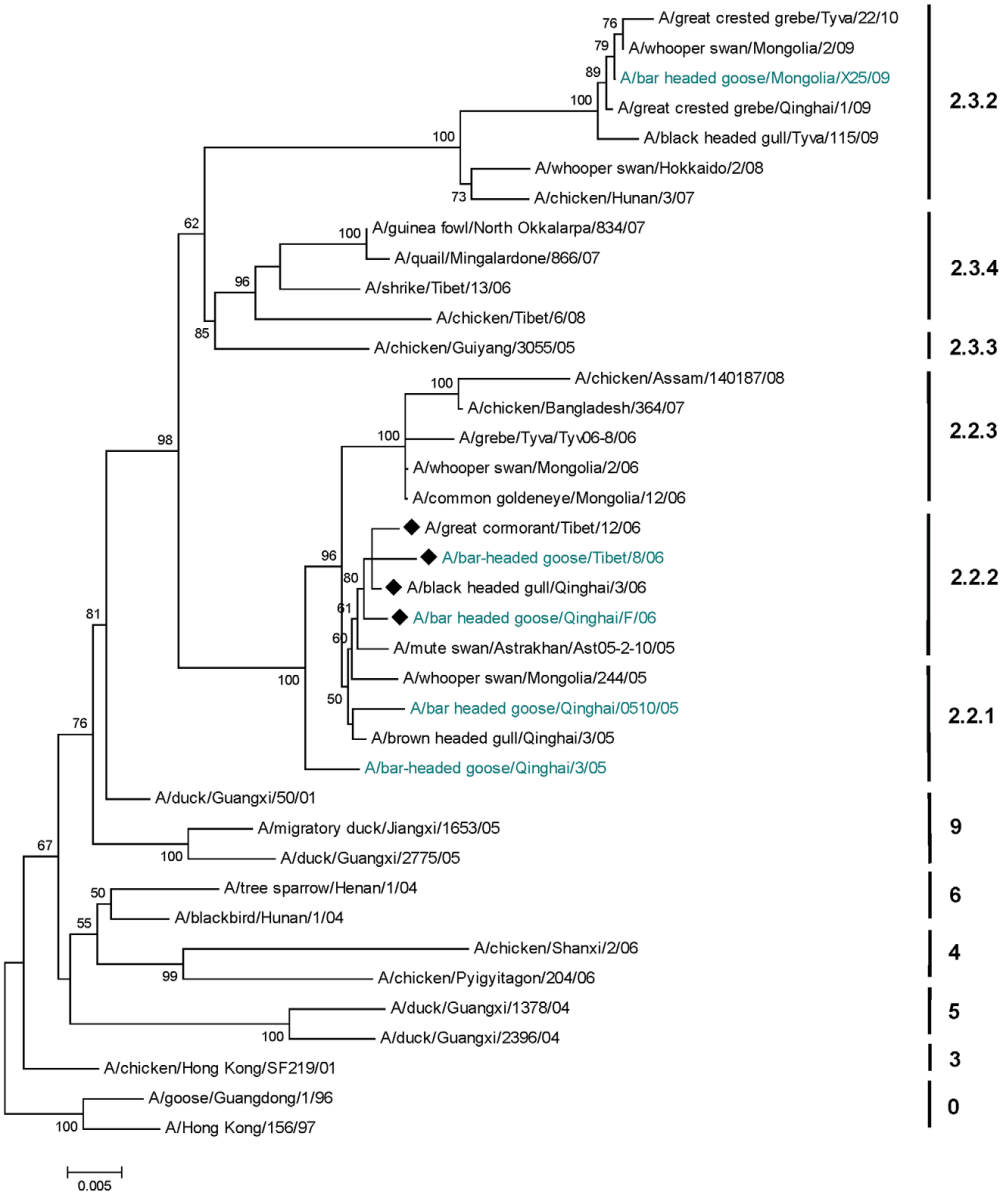
Phylogenetic relationships of HPAI H5N1inferred by neighbor-joining analysis based on 1550 bp fragment of the HA gene. Viruses isolated from the bar-headed goose are highlighted (blue) and the monophyletic grouping of isolates from Tibet and Qinghai are indicated by a symbol (♦).

Phylogenetic analyses indicated that HPAI H5N1 isolates from clades 2.2 and 2.3 infected migratory waterfowl including the bar-headed goose and poultry on the Qinghai-Tibet Plateau. The directionality of virus transmission from domestic to wild birds or vice-versa was however, difficult to ascertain based on the limited number of sequences available within this geographic region. Viruses isolated from bar-headed geese formed distinct sub-clades based on year of outbreak. For instance, isolates from 2005 grouped within sub-clade 2.2.1, and similarly isolates from 2006 and 2008 grouped within sub-clade 2.2.2 and 2.3.2, respectively (Fig. 7). This pattern suggested reintroduction of the virus into bar-headed goose populations, rather than continuous circulation or persistence. Isolates from A/bar-headed goose/Tibet/8/06 and the A bar-headed goose/Qinghai/F/06 formed a monophyletic grouping within sub-clade 2.2.2 and showed high bootstrap support (80%). The evolutionary distance between these two isolates was 0.008 base substitutions per site, lower than the overall average (0.046) for the 38 isolates representative of HPAI H5N1 divergence since 1996.

## Discussion

We hypothesized that if bar-headed geese were a significant contributor in the spread of H5N1 to QHL, certain conditions would have had to occur: (1) exposure of geese to virus sources prior to arriving at QHL, (2) onset of migration and viral shedding before individuals became physiologically compromised, and (3) evidence of genetic relatedness between isolates from virus source regions frequented by geese and QHL. Our findings indicate that these conditions were met; however, we discuss caveats in our findings based on the most current H5N1 disease ecology.

Both the timing of migratory movements and distribution of wintering habitat used by geese around Lhasa exposed them to H5N1 sources (Condition 1) as was evidenced in four ways: (a) location of outbreaks within the BHGO UD (Fig. 2a); (b) detailed movements showing proximity in timing and spatial distribution between marked geese, H5N1 outbreak in chickens, and a captive bar-headed goose farm (Fig. 3 and 4); (c) temporal analysis showing peaks in poultry outbreaks followed by peaks in wild bird outbreaks (Fig. 6); (d) and a significant *a priori* logistic regression between BHGO UD and poultry outbreaks (Table 5^a^). The evidence for goose exposure to H5N1 sources on the Lhasa wintering grounds is consistent. What was unexpected is the discovery of Lhasa as a major wintering area for geese from QHL and the potential of the region as an important H5N1 transmission route – both of which have conservation implications for this species. Prior to this work, little was known about migration of breeding and staging waterbirds at QHL [18], [49]. Of 800 bar-headed goose banding records from the lake in 1987 and 1988, only three returns have been reported (one each in northeast and southwest India [50] and one in Chittagong, southern Bangladesh [51]). Bishop et al. [52] reported that 25% of the global bar-headed goose population winters on the southern Qinghai-Tibet Plateau, however, breeding connectivity for these individuals was previously unknown. Hypotheses regarding the epidemiology of the event at QHL suggested by previous authors [10], [12], [53], [54] were based on inadequate migration data available at the time, and associated models therefore did not examine Tibet as a potential H5N1 transmission link to QHL. For example, Chen et al. [12] and Liang et al. [53] hypothesized that wild birds transported the virus to QHL from Poyang Lake, southeastern China, where 6 apparently healthy wild ducks (species not reported) tested positive for HPAI H5N1. Results from our study and related work at Poyang Lake [55], which examined migration patterns of 9 waterfowl species (n = 62), showed no evidence of migratory connectivity between the 2 lakes located in separate flyways. These findings affirmed the importance for including knowledge of host ecology to inform the debate on wild birds and HPAI transmission.

**Table 5:** Top-ranked Akaike Information Criterion (AIC) models with parameter values for domestic poultry and wild bird H5N1 outbreaks on the Qinghai-Tibet Plateau.

Model^a^	Parameter^b^	*w_i_*	β	SE	z-value	Pr(>|z|)	Pr(>|×^2^|)
Poultry Outbreaks 1	Intercept	0.72	−6.451	2.714	−2.377	0.018	**–**
	PD		0.088	0.042	2.122	0.034	<0.001
	Crop		0.001	0.000	2.167	0.030	<0.001
Poultry Outbreaks 2	Intercept	0.26	11.660	17.780	0.656	0.512	–
	PD		0.078	0.043	1.826	0.068	<0.001
	Crop		0.001	0.000	1.968	0.049	<0.001
	Lat		−0.249	0.494	−0.503	0.615	0.480
	BHGO UD		−10.280	9.393	−1.094	0.274	0.228
Wild Outbreaks 1	Intercept	0.70	−17.330	5.633	−3.076	0.002	**–**
	Lat		0.045	0.166	2.696	0.007	0.004
	Wetl		0.000	0.000	2.222	0.026	0.024
Wild Outbreaks 2	Intercept	0.22	−17.476	5.463	−3.199	0.001	–
	Lat		0.462	0.161	2.860	0.004	0.003

a *A priori* analysis of poultry outbreaks and BHGO UD indicated BHGO exposure to H5N1 sources on the wintering grounds; Single variable *a priori* poultry model: BHGO UD (p = 0.032).

b Parameters for poultry models included bar-headed goose utilization distribution (BHGO UD), poultry density (PD), latitude (Lat), and cropland (Crop). Parameters for wild bird models include BHGO UD, PD, Lat, grassland, and wetlands (Wetl).

*w_i_* = Akaike weights, β = model coefficients, SE = standard error (SE), Pr(>|z|) = significance value, Pr(>|×^2^|) = Chi-square goodness of fit test.

A clear understanding of poultry farming in this remote region is also critical to assessing H5N1 transmission potential. There has been confusion over whether captive bar-headed goose farms exist at QHL, and whether such farms provided introduction of H5N1 virus to this region generally lacking poultry [56]. Through a combination of local investigation, communication with experts, and detailed review of the original blog postings (in Chinese) regarding the farms, we have learned that the farms are not located at QHL, but instead exist 1200 km south of QHL near Lhasa. These conclusions are independently supported by [57]. The largest of the captive bar-headed goose breeding facilities discussed above is included in our study (Fig. 3b and 4), where one of our marked geese spent the winter foraging in the local fields and wetlands.

We found evidence suggesting that migratory movement could occur before the virus impaired a bird's ability to migrate (Condition 2). Recent challenge studies identified the average duration of asymptomatic virus shedding to be 6.5. days in experimentally infected bar-headed geese (A/whooper swan/Mongolia/244/2005H5N1) [58]. Geese from QHL averaged 380 km per day (range 294 km in 1.3 days to 1158 km in 5.1 days; Table 2), indicating a capacity to move the distance between QHL and Lhasa within the asymptomatic period of virus shedding. Migration for most individuals, however, did not occur without time spent at stopover locations, which increased total migration time to an average of one month. Thus, while movement of virus the entire distance (within the asymptomatic period) is possible, as was observed for one bar-headed goose, a more likely mode of transmission is through a relay effect [59]. This would occur if a virus is transported to stopover locations where it can spread among individuals within concentrated areas of feeding and roosting [60], [61] or through environmental persistence [62], [63], and then be forwarded to newly infected individuals along the migratory pathway. We have evidence that stopover locations used by marked geese in this study (Table 1, Fig. 1) are important both for bar-headed geese as well as a number of other important waterbird species (Y.S. Hou, unpubl. data). In addition, recent studies [64], [65] have indicated that mallards *(Anas platyrhynchos)* with previous exposure to homologous LPAI viruses may remain healthy enough to migrate, and this might apply to other waterfowl such as bar-headed geese. The first H5N1 outbreak on the Plateau occurred in chickens in Lhasa in February, 2004 (Table S1), and was followed by the QHL wild bird epizootic in spring of 2005. Unfortunately, none of the seven reported poultry outbreaks have sequences available in open sources such as GenBank. Release of these data would improve our analyses by allowing us to test relationships between isolates from poultry and wild birds from the 2 regions. However, Li et al. [66], in an updated analysis of H5N1 virus evolution in China, includes one sequence from a 2008 outbreak in chickens from a live-bird market (CH/TB/6/08). Here they discovered high sequence similarity between the 2005 QHL epizootic (bar-headed goose isolates, BHG/QH/3/05) and chicken isolates from Tibet in one of the eight H5N1 genes (PB2), supporting a connection between the Lhasa and QHL outbreaks (Condition 3). In addition, our phylogenetic analysis of publicly available sequences included 2 isolates from wild birds in Tibet: a bar-headed goose (A/BHG/TB/8/06) and great cormorant (A/GC/TB/12/06), both from 2006. The analysis indicated that geese were first infected by HPAI H5N1 belonging to clade 2.2 that emerged at QHL during 2005. Since this time, the virus has continued to infect bar-headed geese periodically within the Central Asian Flyway, most recently during the 2009 outbreak in Mongolia. Goose isolates from the QHL and Tibet outbreaks in 2006 formed a monophyletic group within sub-clade 2.2.2 and were closely related, providing additional evidence that migratory birds were agents of transmission between these two outbreak sites in China. Further analysis of isolates in combination with remaining unreleased sequences would help elucidate the route by which viruses moved from southeastern China to QHL including the directionality of virus transmission between poultry and wild birds [8], [10], [12], [48].

Lhasa provides a unique situation for intermixing and potential transfer of disease between wild and domestic birds. Each winter, the Lhasa region experiences increased concentrations of humans, poultry, and wild birds when nomadic herders return to populated centers, chicken production peaks preceding the Chinese New Year festivities in late January [67], and up to 50% of the global population of bar-headed geese winter in sheltered river valleys surrounding Lhasa [52], [68], [69]. H5N1 outbreaks in domestic birds spiked in frequency under such winter conditions [3] followed by an increase in wild bird outbreaks during the spring and breeding seasons (Fig. 6).

The H5N1 situation involving wild birds in the Central Asian Flyway is unique relative to results from studies in the East Asian Flyway (EAF) along the Pacific coast [55], [70]. The EAF boasts some of the world's most productive poultry systems including rice-paddy duck farming in China and parts of Southeast Asia [71]. Whooper swans marked in eastern Mongolia within the EAF demonstrated spatial proximity to poultry outbreaks in Korea and north-eastern China; however, a lack of correspondence in timing and micro-habitat use precluded the likelihood of transmission between the two groups [70]. Eight duck species marked at Poyang Lake in south-eastern China also showed a temporal mismatch between H5N1 outbreaks and arrival of wild ducks to the wintering grounds [55]. Poultry production in the Central Asian Flyway is extensive in the south, particularly in India and Bangladesh, and limited to absent in the central and northern sections (Tibet and northward). H5N1 outbreaks along the flyway mirror human and poultry densities whereby more domestic bird outbreaks occur in the south and wild bird outbreaks in the sparsely populated north [3]. The Qinghai-Tibet Plateau lies within the transition zone along this gradient, and it appears that bar-headed geese may be an important vector in H5N1 spread as evidenced by the size of the infected population at Qinghai Lake [9], [10]. Our study identifies QHL and Lhasa as important linkages between wild and domestic transmission of H5N1 and provides new supporting information regarding the role of wild birds in long distance spread of this virus. Further investigation of wild birds and H5N1 transmission within the Central Asian Flyway will increase our understanding of how wild birds may contribute to virus circulation and the unique pattern of outbreaks in this remote region.

## Supporting Information

Table S1 **Confirmed HPAI H5N1 outbreaks in wild birds and domestic poultry on the Qinghai–Tibet Plateau, 2003–2009.**
(DOC)

Table S2 **PTT performance (as of 1 Sep 2009) for 29 bar-headed geese marked in 2007–2008 at Qinghai Lake, China.**
(DOC)
