# Supplementary material for: Wild Bird Migration across the Qinghai-Tibetan Plateau: A Transmission Route for Highly Pathogenic H5N1
Source: PLoS One. 2011 Mar 9;6(3):e17622. doi: 10.1371/journal.pone.0017622 (PMC3052365; doi:10.1371/journal.pone.0017622)
Supplement: Table S1 — Confirmed HPAI H5N1 outbreaks in wild birds and domestic poultry on the Qinghai–Tibet Plateau, 2003–2009. (DOC) [file pone.0017622.s001.doc]

Table S1. Confirmed HPAI H5N1 outbreaks in wild birds and domestic poultry on the Qinghai – Tibet Plateau, 2003-2009.

| **Outbreak Date** | **Location** | **Type** | **Species** | **Source^a^** |
| --- | --- | --- | --- | --- |
| 2/8/2004 | Lhasa, Tibet | Domestic | Chicken (*Gallus gallus domesticus*) | EMPRES |
| 5/3/2005 | Qinghai Lake, Qinghai | Wild | Bar-headed Goose (*Anser indicus*), Great black-headed Gull (*Larus ichthyaetus*), Brown-headed Gull (*L. brunnicephalus*), and Common Cormorant (*Phalacrocorax carbo*) | EMPRES, MOA |
| 8/10/2005 | Lhasa, Tibet | Domestic | Chicken | MOA |
| 4/23/2006 | Yushu County, Qinghai | Wild | Bar-headed Goose, Ruddy Shelduck (*Tadorna ferruginea*) | EMPRES, MOA |
| 4/23/2006 | Qinghai Lake, Qinghai | Wild | Bar-headed Goose | MOA |
| 5/21/2006 | Guoluo, Qinghai | Wild | Bar-headed Goose, Ruddy Shelduck | EMPRES, MOA |
| 5/21/2006 | Naqu County, Tibet | Wild | Bar-headed Goose, Ruddy Shelduck | EMPRES, MOA |
| 5/26/2006 | Lhasa, Tibet | Wild | Unknown bird spp. | EMPRES |
| 5/26/2006 | Dangxiong District, Tibet | Wild | Bar-headed Goose, Ruddy Shelduck | EMPRES |
| 3/1/2007 | Lhasa, Tibet | Domestic | Chicken | EMPRES, MOA |
| 1/21/2008 | Jiedexu, Gongga County, Tibet | Domestic | Chicken and ducklings | EMPRES, MOA |
| 2/6/2008 | Se Ma, Duilongdeqing County, Tibet | Domestic | Chicken | EMPRES, MOA |
| 3/28/2008 | Zhuba, Basu County, Tibet | Domestic | Chicken | EMPRES, MOA |
| 4/12/2009 | Lhasa, Tibet | Domestic | Poultry | EMPRES, MOA |
| 5/8/2009 | Genggahu Lake, Gonghe County, Qinghai | Wild | Great Crested Grebe (*Podiceps cristatus*), Ruddy Shelduck | EMPRES, MOA |
| 5/27/2009 | Nanhai Prefecture, Qinghai | Wild | Unknown bird spp. | EMPRES |

^a^Outbreak data are from the Chinese Ministry of Agriculture (MOA) and the UN Food and Agriculture Organization's Emergency Prevention System for Transboundary Animal and Plant Pests and Diseases (EMPRES).

**References**

MOA (2009) Ministry of Agriculture of the People's Republic of China, Prevention and control of avian influenza. <http://www.agri.gov.cn/ztzl/fkqlg/yqfb/>

OIE (2010) Update on highly pathogenic avian influenza in animals: Type H5 and H7. [http://www.oie.int/downld/AVIAN%20INFLUENZA/A_AI-Asia.htm](http://www.oie.int/downld/AVIAN INFLUENZA/A_AI-Asia.htm).
