# Supplementary material for: Wild Bird Migration across the Qinghai-Tibetan Plateau: A Transmission Route for Highly Pathogenic H5N1
Source: PLoS One. 2011 Mar 9;6(3):e17622. doi: 10.1371/journal.pone.0017622 (PMC3052365; doi:10.1371/journal.pone.0017622)
Supplement: Table S2 — PTT performance (as of 1 Sep 2009) for 29 bar-headed geese marked in 2007–2008 at Qinghai Lake, China. (DOC) [file pone.0017622.s002.doc]

Table S2. PTT performance (as of 1 Sep 2009) for 29 bar-headed geese marked in 2007-2008 at Qinghai Lake, China.

|  |  |  |  |  |  |  | **Number of Complete Seasons^a^** | | | |
| --- | --- | --- | --- | --- | --- | --- | --- | --- | --- | --- |
| **PTT** | **Age** | **Sex** | **Capture Date** | **Last Trans- mission** | **Working Days** | **GPS Locations** | **Breeding** | **Fall** | **Winter** | **Spring** |
| 67580 | A | F | 3/26/2007 | 6/23/2007 | 90 | 354 | 0 | 0 | 0 | 0 |
| 67582 | A | F | 3/25/2007 | 5/7/2008 | 410 | 2564 | 1 | 1 | 1 | 1 |
| 67586 | A | M | 3/26/2007 | 10/9/2007 | 198 | 539 | 1 | 0 | 0 | 0 |
| 67690 | A | F | 3/25/2007 | 1/12/2008 | 294 | 1849 | 1 | 1 | 0 | 0 |
| 67693 | A | F | 3/26/2007 | 6/7/2007 | 74 | 123 | 0 | 0 | 0 | 0 |
| 67695 | A | M | 3/25/2007 | 3/21/2009 | 728 | 4624 | 2 | 2 | 2 | 1 |
| 67698 | A | F | 3/31/2007 | 2/7/2008 | 314 | 1507 | 1 | 1 | 0 | 0 |
| 67699 | A | F | 3/26/2007 | 11/4/2007 | 224 | 938 | 1 | 0 | 0 | 0 |
| 74898 | A | F | 3/30/2007 | 4/2/2008 | 370 | 1794 | 1 | 1 | 1 | 0 |
| 74899 | A | M | 3/30/2007 | 10/23/2007 | 208 | 682 | 1 | 0 | 0 | 0 |
| 74900 | J | F | 3/31/2007 | 4/1/2008 | 368 | 1670 | 1 | 1 | 1 | 0 |
| 74901 | J | M | 3/31/2007 | 11/23/2008 | 604 | 3610 | 2 | 2 | 1 | 1 |
| 74902 | A | F | 3/30/2007 | 11/15/2008 | 597 | 3247 | 2 | 1 | 1 | 1 |
| 74903 | A | M | 3/31/2007 | 4/29/2007 | 30 | 84 | 0 | 0 | 0 | 0 |
| 82076 | J | F | 3/31/2008 | 11/15/2008 | 230 | 1071 | 1 | 1 | 0 | 0 |
| 82077 | A | F | 4/3/2008 | 4/11/2008 | 9 | 86 | 0 | 0 | 0 | 0 |
| 82078 | A | M | 4/2/2008 | 4/9/2008 | 8 | 65 | 0 | 0 | 0 | 0 |
| 82079 | A | M | 4/2/2008 | Active | 518 | 2366 | 1 | 1 | 1 | 1 |
| 82080 | A | M | 4/2/2008 | 4/30/2009 | 394 | 3003 | 1 | 1 | 1 | 0 |
| 82081 | A | M | 4/1/2008 | Active | 519 | 3084 | 1 | 1 | 1 | 1 |
| 82082 | A | F | 3/30/2008 | Active | 521 | 3490 | 1 | 1 | 1 | 1 |
| 82083 | J | M | 3/30/2008 | 4/26/2008 | 28 | 93 | 0 | 0 | 0 | 0 |
| 82084 | A | M | 3/30/2008 | Active | 521 | 2236 | 1 | 1 | 1 | 1 |
| 82085 | A | M | 3/30/2008 | 4/1/2009 | 368 | 2161 | 1 | 1 | 1 | 0 |
| 82086 | A | F | 3/31/2008 | Active | 520 | 2016 | 1 | 1 | 1 | 1 |
| 82087 | A | M | 4/3/2008 | 10/12/2008 | 193 | 607 | 1 | 0 | 0 | 0 |
| 82088 | A | M | 3/28/2008 | 5/20/2008 | 54 | 27 | 0 | 0 | 0 | 0 |
| 82089 | A | M | 3/29/2008 | 4/5/2008 | 8 | 21 | 0 | 0 | 0 | 0 |
| 82090 | A | M | 4/3/2008 | 10/28/2008 | 209 | 1110 | 1 | 0 | 0 | 0 |
|  |  |  |  |  | **x= 297** | **45,021** | **21** | **15** | **13** | **9** |

^a^Brownian bridge and home range analyses were divided into breeding (23 May - 26 Sep), fall migration (27 Sep - 9 Dec), winter (10 Dec - 5 Mar), and spring migration (6 Mar - 22 May). Analyses include seasons from the first annual cycle for each individual.
